# Deep-sea Ordovician lingulide brachiopods and their associated burrows suggest an early colonization of proximal turbidite systems

**DOI:** 10.1038/s41598-023-49875-8

**Published:** 2023-12-20

**Authors:** Maximiliano Paz, M. Gabriela Mángano, Luis A. Buatois, Debora M. Campetella, Colin Sproat, Manuel Pérez-Pueyo, Laura Piñuela, José Carlos García-Ramos

**Affiliations:** 1 Department of Geological Sciences, University of Saskatchewan, 114 Science Place, Saskatoon, SK S7N 5E2 Canada; 2 Instituto de Investigación en Paleobiología y Geología, Universidad Nacional de Río Negro, General Roca, Argentina; 3 Instituto de Investigación en Paleobiología y Geología (IIPG), Consejo Nacional de Investigaciones Científicas y Técnicas (CONICET), Av. J. A. Roca 1242, 8332 General Roca, Argentina; 4 Grupo Aragosaurus-Instituto Universitario de Investigación en Ciencias Ambientales de Aragón (IUCA), Departamento de Ciencias de La Tierra, Facultad de Ciencias, Universidad de Zaragoza, 50009 Zaragoza, Spain; 5 Museo del Jurásico de Asturias (Jurassic Museum of Asturias), Rasa de San Telmo, 33328 Colunga, Spain

**Keywords:** Palaeontology, Sedimentology

## Abstract

Trace fossils from Ordovician deep-marine environments are typically produced by a shallow endobenthos adapted to live under conditions of food scarcity by means of specialized grazing, farming, and trapping strategies, preserved in low-energy intermediate to distal zones of turbidite systems. High-energy proximal zones have been considered essentially barren in the early Paleozoic. We report here the first trace and body fossils of lingulide brachiopods in deep-marine environments from an Upper Ordovician turbidite channel-overbank complex in Asturias, Spain. Body and trace fossils are directly associated, supporting the interpretation of a lingulide tracemaker. Ellipsoidal cross-section, cone-in-cone spreite, and spade morphologies suggest the specimens belong to *Lingulichnus verticalis*. The oblique orientation in both trace and body fossils is the result of tectonic deformation. The organisms were suspension feeders showing escape, dwelling, and equilibrium behaviours controlled by sedimentation rates associated with turbidite deposition. These trace fossils and their in situ producers represent the oldest evidence of widespread endobenthos colonization in high-energy, proximal areas of turbidite systems, expanding the bathymetric range of *Lingulichnus* and the variety of behaviours and feeding styles in early Paleozoic deep-marine environments.

## Introduction

Paleozoic and modern Lingulida (Brachiopoda) occur in a wide variety of environments, ranging from nearshore to bathyal, although they are common in intertidal, shoreface, and offshore environments^1–13^. An endobenthic lifestyle in lingulides has been established since the Early Ordovician (Cambrian endobenthic evidence remains contested^14–17^). This mode of life has been instrumental to avoid physiochemical stresses at the sediment surface in intertidal settings, protecting the organisms from physical reworking, drying out during low tide conditions, and predation^1,6,14,18,19^. The many adaptations lingulides exhibit provide them with a high resilience to a wide range of environments characterized by variations in salinity, temperature, pH, oxygen, and bathymetry^2,4,20,21^. Hence, these organisms are considered generalists, and are often seen as “disaster taxa” or ecological opportunists that dominated benthic communities of marginal- and shallow-marine environments in the aftermath of mass extinctions, such as during the Early Triassic^11,21–26^.

Lingulide burrows are known as *Lingulichnus*^27–29^, a trace fossil reflecting dwelling, equilibrium, and escape behaviours depending on the sedimentation rate and hydrodynamic energy of depositional events^30–32^. *Lingulichnus* is a trace fossil typical of ancient tidal flat, deltaic, estuarine, and shoreface successions, with some specimens recorded in offshore and lagoonal deposits^14,19,30,33–47^. Until now, *Lingulichnus* has never been found in ancient deep-marine successions^11^.

Deep-marine environments are typically characterized by several stress factors that restrict benthic colonization, such as an extremely low food supply owing to the high bathymetry and consequent intense degradation process in the water column^48^. Therefore, a special type of trace fossils known as graphoglyptids (e.g., *Cosmorhaphe, Paleodictyon, Megagrapton*) can be found in great abundances at the base of deep-sea turbidites (constituting pre-depositional biogenic structures), reflecting the predominance of specialized farming and trapping behaviours, which together with grazing traces, are associated with optimized resource exploitation^48–63^. Overall, suspension-feeding trace fossils are rare in deep-marine turbidite settings^48,60^, although isolated vertical burrows that may be interpreted as produced by suspension feeders or passive predators have been recorded in modern abyssal plain deposits (e.g. *Skolithos*^64,65^). Whereas graphoglyptids are preserved in deposits formed in areas of relatively low energy within the turbidite system (representing the *Paleodictyon* ichnosubfacies of the *Nereites* Ichnofacies), high-energy zones such as channel-levee complexes and proximal areas of turbidite splays contain deposits that tend to be less bioturbated. These high-energy zones of the turbidite system are dominated by burrows attributed to callianassid shrimps in the *Ophiomorpha rudis* ichnosubfacies of the *Nereites* Ichnofacies, commonly occurring in channel and proximal lobe facies^52,57,66–69^. However, such trace-fossil assemblage was not established in deep-marine environments until the Late Jurassic^68^. Early Paleozoic high-energy proximal zones are typically unbioturbated or show very low levels of bioturbation and few trace fossils^70^.

**Figure 1:**
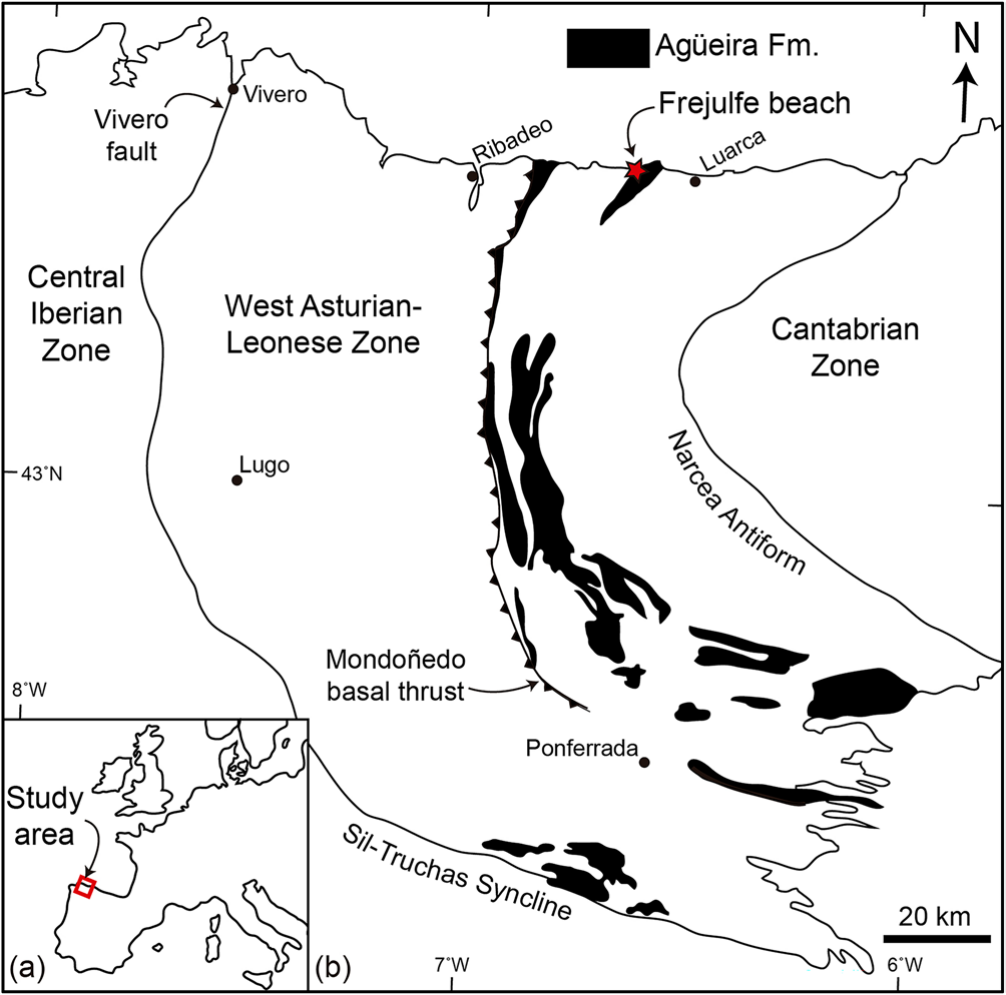
Location map of the study area. (**a**) Location of the West Asturian-Leonese Zone (WALZ) within Western Europe. (**b**) Location of the study area (Frejulfe beach) within the WALZ. The map shows the extent of the Agüeira Formation (black), relevant tectonic structures, and regional geological zones. Map generated using data from Pérez-Estaún and Marcos^84^ and Pérez-Estaún et al*.*^73^ and modified by M.P. in Adobe Illustrator CS6.

We report here the trace fossil *Lingulichnus verticalis* associated with lingulides in life position from an Upper Ordovician deep-marine turbidite channel and overbank complex in Asturias, Spain (Frejulfe beach, Fig. 1a). The objectives of this study are to: (1) document the lingulides and their trace fossils from these deep-marine deposits, and (2) discuss the implications of this finding from the perspective of the early Phanerozoic colonization of the deep sea. The report of lingulide trace fossils expands the range of behaviours and feeding techniques in ancient deep-marine successions and the bathymetric range of *Lingulichnus* and represents the oldest record of widespread colonization of relatively high-energy, proximal turbidite deposits.

## Geological setting

The study area is part of the West Asturian-Leonese Zone (WALZ), a Variscan tectonostratigraphic domain of the Paleozoic Iberian Massif of Spain^71,72^. The WALZ is limited to the west by the Vivero Fault, to the SW by the Sil-Truchas syncline, and to the east by the Narcea Antiform (Fig. 1b^73^). The stratigraphy of the WALZ consists of Precambrian to Carboniferous rocks, but is dominated by *ca*. 11 km-thick Cambrian-Ordovician rocks, which in the coastal area can be subdivided into the Cándana Group heterolithic siliciclastic rocks (Precambrian to lower Cambrian), Vegadeo Formation limestones (lower to middle Cambrian), Los Cabos Group heterolithic siliciclastic rocks (middle Cambrian to Lower Ordovician), Luarca Formation shales (Lower to Upper Ordovician), Agüeira Formation heterolithic siliciclastic rocks (Upper Ordovician), and the Vega de Espinareda quartzites (Upper Ordovician^73,74^). This thick stratigraphic succession was a product of fault-controlled, rapidly subsiding troughs associated with extensional tectonic activity in the shelf of the northern margin of Gondwana^73–78^. Volcanic activity associated with the extensional regime is represented by the older to partially coeval Castro Formation of the Cantabrian zone^79^. Ordovician paleogeographic data from climate indicators and paleomagnetic evidence suggest a high-latitude position for the study area in the proximity of a glaciated area^76,78,80^.

The studied interval (Frejulfe beach, Fig. 1b) is part of the Agüeira Formation, a 300–3000 m-thick turbiditic metasandstone, sandstone, siltstone, slate, and mudstone succession. Its age is indicated by stratigraphic relationships and the presence of rare brachiopods, trilobites, bryozoans, echinoderms, and gastropods, suggesting that the bulk of sedimentation corresponds to the Katian with the uppermost levels probably reaching the lower Llandovery^73,76,78,81–85^. In the study area (Navia-Alto Sil Domain), this formation has been described as a 1300–1500 m-thick succession of very thin- to thick-bedded, very fine-grained sandstone and mudstone^73,82,83^. Sedimentary structures in the sandstone include normal grading, parallel lamination, climbing- and current-ripple cross lamination, convolute lamination, and slumps^82^. Paleocurrents are consistently towards the N and NE and suggest axial transport in an elongated trough^77,82,83^. The Agüeira Formation was interpreted as Bouma turbidites formed in a submarine fan with development of proximal, inner fan facies towards the South and transitioning to a surrounding basin plain towards the North^81–84^. The sandstone-mudstone succession shows several fining- and thinning-upward cycles interpreted as the result of fault block uplift in source areas^83^. Trace fossils in the study area are relatively rare, and include *Arenicolites*, *Cosmorhaphe* (probably *Helminthopsis*), *Granularia*, *Helminthopsis*, *Protopaleodictyon*, and *Spirophycus*^83^. Only line drawings of these trace fossils were recorded in literature, and hence, checking their determinations is not possible, other than pointing that *Granularia* is no longer regarded as a valid ichnotaxon^86^. Outside of the study area, *Helminthopsis*, *Planolites*, *Protopaleodictyon*, *Taphrhelminthopsis*, and inclined burrows have been mentioned^84^. The only illustrated ichnotaxon has been referred to *Taphrhelminthopsis.* Paleozoic records of this ichnogenus are now referred to *Psammichnites*^87^, but the diagnostic characteristics of this ichnotaxon are not seen in the photograph provided, and therefore, the existence of this trace fossil in the formation cannot be confirmed.

## Results

### Sedimentary deposits

**Figure 2:**
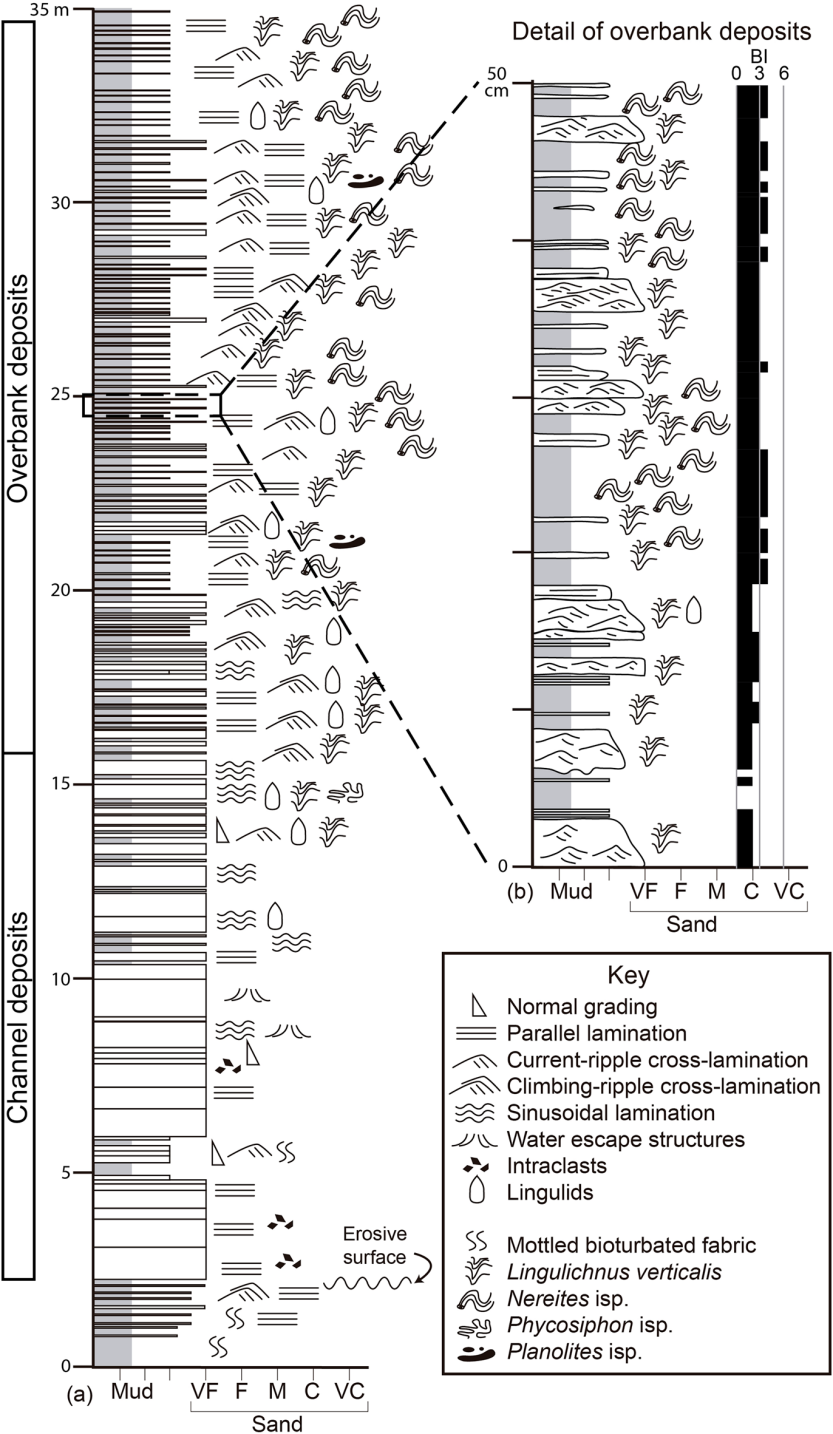
Studied section of the Agüeira Formation in the Frejulfe beach, near Puerto de Vega, Asturias, Spain. (**a**) Section displaying the fining-upward pattern of the channel and overbank deposits. (**b**) Detail of overbank deposits, with the quantification of bioturbation index (BI). Created by M.P. in Adobe Illustrator CS6.

**Figure 3:**
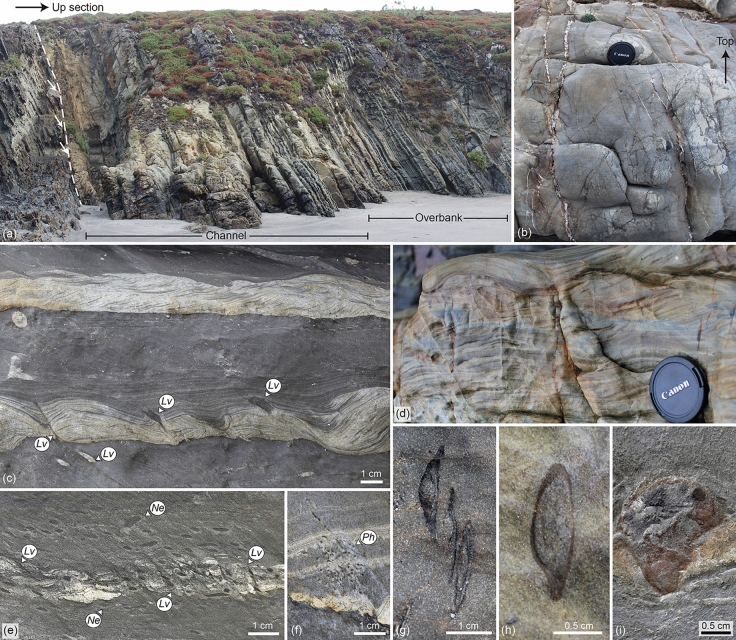
Sedimentary deposits, body fossils, and trace fossils of the described Agüeira Formation succession. (**a**) Outcrop view of the studied section showing the extent of channel and overbank deposits. An erosive surface is observed at the base of the channel deposits (dashed line), truncating a mud-rich succession below (white arrows). The whole outcrop photograph is *ca.* 25 m long. (**b**) Thick-bedded, parallel- to slightly sinusoidal-laminated, very fine-grained sandstone of the channel deposits. Lens cap is 58 mm diameter (interval at 6.5 m of the section). (**c**) Climbing-ripple to current-ripple cross-laminated very fine-grained sandstone to siltstone with parallel-laminated siltstone towards the top, interbedded with mudstone. *Lingulichnus verticalis* (*Lv*) is delineated. Note a spade morphology in the middle specimen (interval at 16.5 m of the section). (**d**) Thick-bedded, climbing-ripple cross-laminated to sinusoidal-laminated, very fine-grained sandstone. Lens cap is 58 mm (interval at 21.25 m of the section). (**e**,**f**) *Nereites* isp. (*Ne*), *Phycosiphon incertum* (*Ph*), and *Lingulichnus verticalis* (*Lv*), in parallel-laminated siltstone and mudstone (photographs from loose block). (**g**,**h**) Lingulide brachiopods with unaltered calcium phosphate shells (interval at 13.2 m of the section). (**i**) Lingulide brachiopod replaced by pyrite and iron oxides (photograph from loose block). Photographs taken by M.P. and labels created by M.P. in Adobe Illustrator CS6.

The described succession can be subdivided into two types of deposits, namely channel and overbank deposits of a deep-marine environment (Fig. 2). The entire section consists of a fining- and thinning-upward stacking pattern, with the channel deposits occurring towards the lower part of the section above an erosive surface (Figs. 2, 3a), whereas the overbank deposits can be found in the upper part separated by a gradual transition from the underlying channel deposits.

The channel deposits consist of 10–100 cm-thick, massive, and parallel- to sinusoidal-laminated, very fine-grained sandstone, either amalgamated or interbedded with 1–3 cm-thick mudstone (Fig. 3b). Rarely, normally graded and current-ripple cross-laminated, very fine-grained sandstone occurs. Soft-sediment deformation structures include load casts, flame structures, and large (25–50 cm thick) water escape structures. The sandstone shows sharp or loaded bases, locally with pebble-sized, rounded intraclasts. The tops of beds are sharp, and some contain 30–50 cm wide carbonate concretions. The mudstone caps the sandstone with transitional bases and sharp to loaded tops. At a large scale, the deposits display an erosive surface scouring *ca*. 1 m of the underlying succession, and tabular and rarely lenticular geometry (although the lateral extension of the outcrop is limited, usually < 10 m). Bioturbation index is 0 in the thicker-bedded intervals, and 1–2 in the thinner-bedded intervals, containing a few specimens of *Phycosiphon incertum* and *Lingulichnus verticalis*. Lingulide brachiopods occur within the thinner sandstone beds.

The existence of a basal erosive surface, fining- and thinning-upward stacking pattern, predominance of high-density, Bouma T_a_ (massive sandstone) and T_b_ (parallel-laminated sandstone) turbidites, and thick bedding suggest that these deposits represent a channel environment^88–90^. Sinusoidal lamination indicates bedload transport and high rates of suspended load fallout associated with rapid deposition in a channel^91^.

The overbank deposits comprise 1–20 cm-thick, climbing-ripple to current-ripple cross-laminated, and parallel-laminated, very fine-grained sandstone to siltstone, interbedded with mudstone of variable thickness (Fig. 3c,d). Locally, the sandstone and siltstone have sinusoidal lamination or normal gradation, whereas the mudstone shows mm-thick, very fine-grained sandstone to siltstone laminae. The thinner sandstone to siltstone beds display climbing-ripple or current-ripple cross-lamination, and normal grading to parallel-lamination towards the top. The thicker beds can be stacked into bedsets of two to four beds and mostly comprise climbing-ripple cross-laminated sandstone. Bases are typically loaded and bioturbated, and rarely erosive. Tops are usually bioturbated. Flame structures are common within the sandstone to siltstone beds. At a large scale, the succession shows a fining- and thinning-upward pattern, transitioning from intervals with a mud/sand ratio of 1 at the lower part, to 5 towards the top. Bioturbation index is 1–4 in the thinner beds, 0 in the thicker beds, and 3–5 in the mudstone. Trace fossils include *Nereites* isp.*, Phycosiphon incertum, Planolites* isp., and *Lingulichnus verticalis* (Fig. 3c,e,f). Although all trace fossils can be found in both lithologies, *Nereites* isp. is preferentially preserved in the mudstone, and *Lingulichnus* is usually observed in the sandstone to siltstone (Fig. 2b). Lingulide brachiopods are found in the thicker beds.

The predominance of low-density, Bouma T_cde_ turbidites (rippled and parallel-laminated sandstone to siltstone, and mudstone), their presence right above turbidite channel deposits, and the recurrence of climbing ripples associated with high aggradation due to flow expansion (typical of channel margins affected by flow stripping) indicate an overbank environment^88,90,91^. The lack of slumped horizons in the Frejulfe succession precludes the interpretation of a levee environment^92,93^.

The whole succession in the study area shows a clear direction of cleavage inclined 35-45º with respect to stratification. Moreover, thin section analysis indicates that clay minerals in mudstone are preferentially oriented in the direction of cleavage, supporting the cleavage orientation observed in outcrops. Drag folds and inverse faults measuring decimeters to centimeters also occur, indicating an extension strain direction similar to the cleavage plane.

### The lingulide body fossils

Lingulide brachiopods are common in the studied succession of the Agüeira Formation, usually observed in cross-section on rock faces, either preserved as unaltered calcium phosphate shells (Fig. 3g,h), or as poorly preserved pyritized replacement that only preserve the gross outline of the shell and lack much surface detail (Fig. 3i). The lingulide shells are preferentially preserved in the thinner sandstone to siltstone beds of the channel deposits and in the thicker sandstone beds of the overbank deposits. No complete, conjoined shells are yet known from the formation and surface or crack out collection of shells is difficult due to the well-consolidated nature of the rock. Moreover, the siliciclastic composition of the rock makes acid dissolution extraction methods unviable. The lack of well-preserved fossil material makes a complete systematic description and confident assignment of the shells to known lingulide taxa difficult to impossible but a few remarks on their preserved morphology follow.

The unaltered calcium phosphate shells are approximately 10–20 mm in length and 3–10 mm in width, elliptical in outline, and approximately twice as long as wide and biconvex in lateral profile (Fig. 3g,h). The shell surface appears to be smooth, but the presence of growth lines or other types of surface ornamentation is difficult to determine in cross-section. Pedicle openings are visible in some shells (e.g. Fig. 3h) both in transverse cross section and lateral profiles. The absence of muscle scars and other internal shell features precludes a precise taxonomic assignment. However, many of the shells exhibit the characteristic biconvex shape and teardrop shaped outline of the linguliformean order Lingulida Waagen, 1885 and superfamily Linguloidea Menke, 1828. The elliptical outline of some of the shells and indications of a well-developed ventral pseudointerarea (Fig. 3g,i) suggest affinities with the Obolidae King, 1846, but the lack of diagnostic features in these specimens does not allow for definitive identification beyond the superfamily level. Lingulides have never been reported from the Agüeira Formation, only the brachiopods *Aegiromena, Rafinesquina,* and *Svobodaina* have been mentioned but never figured^73,81^.

The pyritized specimens are slightly larger and are in most cases replaced by iron oxides (Fig. 3i). Therefore, they range from 15 to 55 mm in length and 10–25 mm in width, yet in many cases the replacement distorts the original shell shape. Like the unaltered shells, they have an elliptical outline and are approximately twice as long as they are wide. The replacement has destroyed any indication of surface ornamentation on the shells. No indications of muscle field are preserved in these specimens despite better preservation of their three-dimensional shape. The biological affinities of these specimens are not as certain given the unusually large size for the specimen and poor preservation. Many are similar in shape to the unaltered shells (e.g. Fig. 3i); however, some are much more amorphous in appearance and may represent the effects of amorphous pyritized organic matter or be the result of other diagenetic processes.

Both forms are commonly associated with burrows (see below). Shells are usually larger than the size range of lingulide trace fossils (see below), perhaps reflecting a taphonomic bias resulting in the preferential preservation of larger and more robust adult body fossils over relatively small fragile juvenile shells^31^.

### The lingulide trace fossils

**Figure 4:**
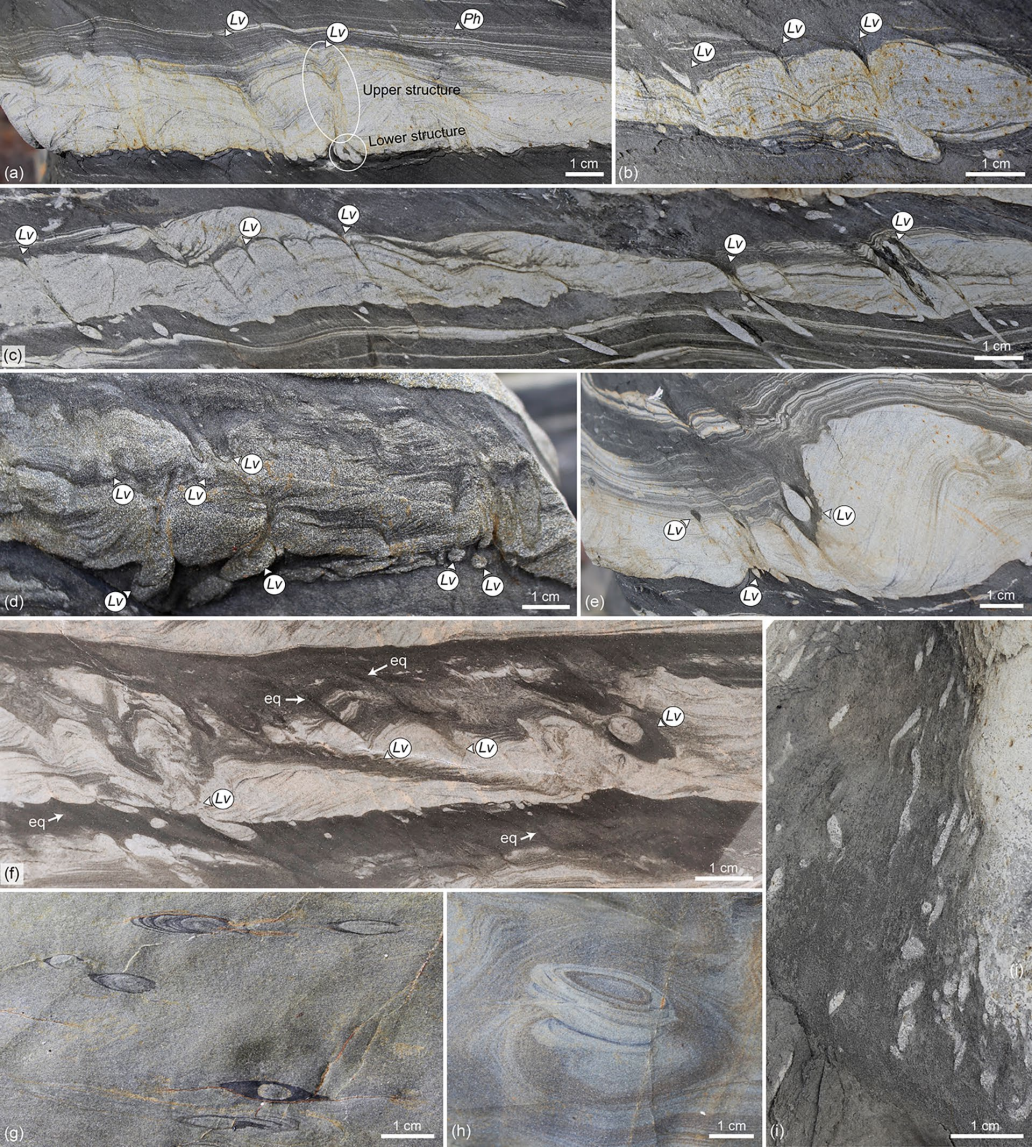
Cross-section and plan views of *Lingulichnus verticalis* of the Agüeira Formation. (**a**,**b**) *Lingulichnus verticalis* (*Lv*) in current-ripple cross-laminated siltstone. *Phycosiphon incertum* (*Ph*) occurs towards the top of the bed. The lower and upper structure of the trace fossil are delineated (photograph from loose block). (**c**) *Lingulichnus verticalis* (*Lv*) in current-ripple cross-laminated siltstone showing a variety of shapes in its lower structure, such as almond (left) and tube (right, photograph from loose block). (**d**) Intra-bed and basal colonization surfaces of *Lingulichnus verticalis* (*Lv*, interval at 18.4 m of the section). (**e**,**f**) Occurrences of *Lingulichnus verticalis* (*Lv*) showing the passively infilled burrow and muddy burrow boundary, in current-ripple cross-laminated very fine-grained sandstone to siltstone. Note equilibrium spreite (eq) within the interbedded mudstone, interpreted as slow upward migration during periods of low sedimentation rate (interval at 18.4 m of the section). (**g**,**h**) Top plan views of the upper structure in medium-bedded sandstone, displaying the typical ovoid shape with pointed and rounded edges in the burrow, and concentrical views of the cone-in-cone spreite around it (interval at 13.2 m of the section). (**i**) Top plan views of *Lingulichnus verticalis* associated with thin-bedded, current ripple cross-laminated siltstone (photograph from loose block). Photographs taken by M.P. and labels created by M.P. in Adobe Illustrator CS6.

**Figure 5:**
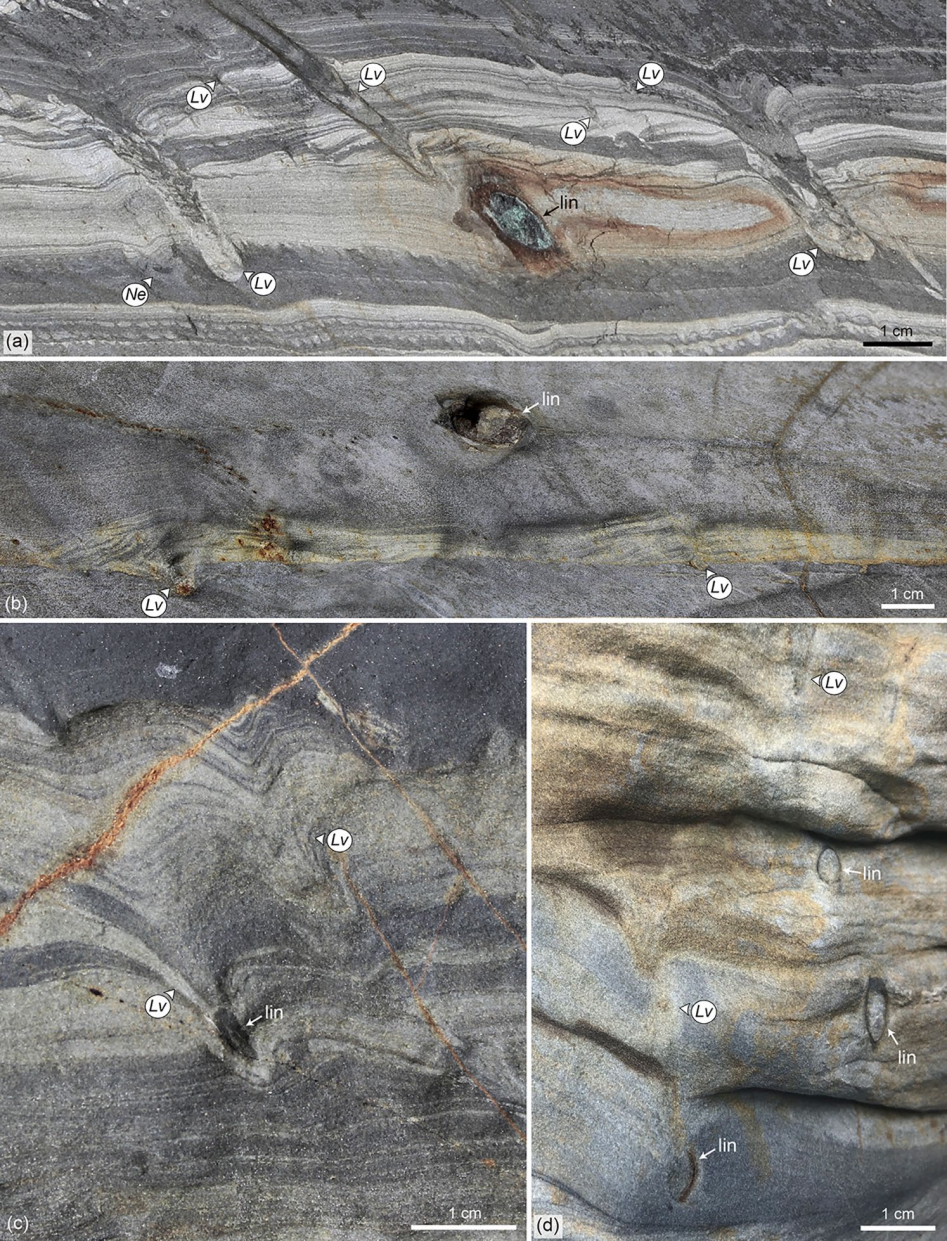
Examples of *Lingulichnus verticalis* preserved in association with lingulides. (**a**,**b**) Lingulide brachiopod (lin) laterally and vertically associated with *Lingulichnus verticalis* (*Lv*). In A, the body fossil occurs within thin-bedded, current ripple cross-laminated very fine-grained sandstone to siltstone, whereas in B the lingulide is observed in parallel-laminated mudstone (photographs from loose block)*.* (**c**,**d**) Lingulide brachiopod (lin) occurring at the base of *Lingulichnus verticalis* (*Lv*)*,* within thick-bedded, current-ripple cross-laminated, and parallel-laminated very fine-grained sandstone (interval at 16.5 m and 21.25 m of the section, respectively). Photographs taken by M.P. and labels created by M.P. in Adobe Illustrator CS6.

The most common lingulide trace fossils in the succession can be assigned to *Lingulichnus verticalis* Hakes, 1976. They consist of oblique (35-45º of inclination) to rarely vertical, passively filled burrows comprising a lower rounded to ellipsoidal structure, and an upper, straight, tubular structure surrounded by concave-up spreite (Fig. 4a–i). Ovoid to oval plan views have rounded and pointed edges on either side. Most burrows are tilted towards the direction of cleavage, which typically matches the direction of paleocurrent (provided by current-ripple cross-lamination, Figs. 4a–f, 5a–d). Detailed close-up photographs of the trace fossil can be found in Supplementary Fig. S1.

The lower structure is typically rounded to ellipsoidal, but also rarely shows tubular, cone, almond, spade, upside-down heart, and “J” morphologies (Figs. 3c, 4a,c, Supplementary Fig. S1). This structure is 1–20 mm wide and 1–10 mm high, whereas tubular forms are 1–5 mm wide and 5–100 mm long. When elongated, this structure is oriented oblique, in the same direction than the upper part.

The upper structure is oblique to vertical, with a straight course, displaying either a passively infilled cone-in-cone spreite with its causative burrow (Fig. 4b,e,f) or only the spreite (Fig. 4a,c,d, Supplementary Fig. S1). The causative burrow has tube-, funnel-, cone-like, and rarely spade morphologies (Fig. 3c). It is filled by sandstone, siltstone, and/or mudstone, and locally shows a 1–4 mm-thick, muddy burrow boundary (Fig. 4e–g). It is 1–5 mm wide and 10–65 mm long (usually constrained by the thickness of the turbidite bed). Plan views of the burrow are hard to find because of its oblique orientation; however, in many instances, an ovoid, ellipsoidal and rarely circular shape occur, measuring 1–5.5 mm wide and 1.5–15 mm long (Fig. 4g–i). The spreite is represented by stacked, concave-up (retrusive) and rarely concave-down (protrusive) laminae, which can be preserved around the burrow or below. The spreite is 1–13 mm diameter and 4–65 mm long.

The lower structure is commonly preserved as full-reliefs in mudstone (hemipelagite) below sandstone or siltstone (turbidite), or at mudstone-sandstone or mudstone-siltstone contacts, most of the time in cross-section. Rarely, it can be observed within sandstone or siltstone (turbidite), as intra-bed colonization (Fig. 4d). The upper structure is preserved as full reliefs, crosscutting the sandstone or siltstone (turbidite). In some instances, the upper spreite is extremely long extending through the interbedded mudstone and crosscutting a few sandstone or siltstone intervals (Fig. 4f). The vertical extension of these specimens could reach 25 cm. *Lingulichnus verticalis* usually occurs in clusters of 5–20 specimens per bed, although isolated specimens are also common.

Lingulides have been found preserved in direct association with the trace fossil. In most instances, lingulides occur in the same intervals than *Lingulichnus verticalis*, namely very thin- to thin-bedded (< 10 cm-thick), current- and climbing-ripple cross-laminated siltstone, showing a direction of inclination towards the direction of cleavage and paleocurrent (like the inclination of *Lingulichnus verticalis*, Figs. 4a–i, 5a,b). In a few examples, thin- to medium-bedded (> 5 cm-thick), current- and climbing-ripple cross-laminated sandstone to siltstone contain lingulides preserved below the spreite of *Lingulichnus verticalis* with their anterior end facing up (Fig. 5c,d).

Tube-like burrows with ellipsoidal bedding plane cross-section surrounded by cone-in-cone spreite and the existence of spade morphologies indicate that this trace fossil can be attributed to *Lingulichnus* Hakes, 1976. Burrow inclination in *Lingulichnus* has been interpreted as oblique shell orientation to enhance pseudosiphon respiration in overpopulated occurrences, and the ichnospecies *Lingulichnus inclinatus* has been erected^30^. However, preferential burrow inclination to currents has never been reported from modern lingulides^4^, possibly due to the close position of both inhalant and exhalant currents. Moreover, in the present specimens, burrow inclination coincides with the cleavage plane, suggesting that tilting resulted from tectonic deformation^94^. Virtual deformation of images using the cleavage angle generates near vertical *Lingulichnus* and ripple slope angles closer to a regular angle of repose (e.g., Figs. 3c, 4e). This fact and the existence of vertical *Lingulichnus* in undeformed, relatively thick sandstone beds (Fig. 5d) indicates the trace fossil might have been vertical before deformation and supports an assignment to *Lingulichnus verticalis*. J-shaped tubes suggest affinities with *L. hamatus* Zonneveld and Pemberton, 2003, yet more specimens are needed to support this designation. *Lingulichnus verticalis* is typical of Cambrian to Cenozoic tidal flat and shoreface environments, but it has also been observed in lagoonal and offshore deposits^2,14,19,28,30,33,36,37,41,42,95^.

*Lingulichnus* represents the dwelling, equilibrium, and escape structure of lingulide brachiopods^27,28,30^. In the present study, the occurrence of lingulide body fossils in lateral association and within *Lingulichnus* supports the interpretation of these organisms as the trace fossil producers. Lingulides are endobenthic suspension feeders that dwell in the sediment with the anterior end of the shells pointing upwards, and with the pedicle at the posterior providing anchor to the bottom of the burrow by a mucous secretion^2,96^. The anterior end is at the sediment–water interface or slightly above and contains the pseudosiphons that are used for water intake and output. This allows for suspension feeding using the lophophore protected within the mantle cavity between the shells. The organisms can move up and down within their burrow by means of the retraction and protraction of the pedicle^2,96^. Initial burrowing from the surface is produced with the pedicle oriented downward in the sediment in Obolidae (as was likely the case in the present specimens), whereas burrowing in a U-shaped trajectory with the pedicle trailing behind the shell is common in the Lingulidae family^18,96,97^.

In the studied specimens, the lower structure of *Lingulichnus* is equal in size or larger than the upper structure, even when it shows tube-like morphology, suggesting that it represents a (turbidite) cast of the shell burrow rather than a cast of the pedicle burrow. In the case of rounded, ellipsoidal, and almond shapes, the sedimentation event probably casted the shell trace in a shallow endobenthic position, whereas the tube shapes represent the casting of an organism in a deeper endobenthic position. The cone and spade morphologies in the upper structure are associated with rare instances in which both the shell and pedicle were casted. Well-defined morphology in the lower structure suggests emplacement in more cohesive sediment, whereas the irregular morphologies are associated with deformation, probably due to sediment being disturbed by the contracting movement of the pedicle while moving upwards.

The upper structure can be interpreted in different ways. In the cases where the passively infilled burrow is preserved with the surrounding cone-in-cone spreite, it is regarded as recording the upward migration of the producer and the cast of the shell burrow within the turbidite event. Where only the spreite is preserved, the trace fossil was probably produced by a rapid upward migration, and the new colonization surface may have occurred within a mudstone interval above the turbidite event, where the lack of sediment contrast precludes burrow visualization.

**Figure 6:**
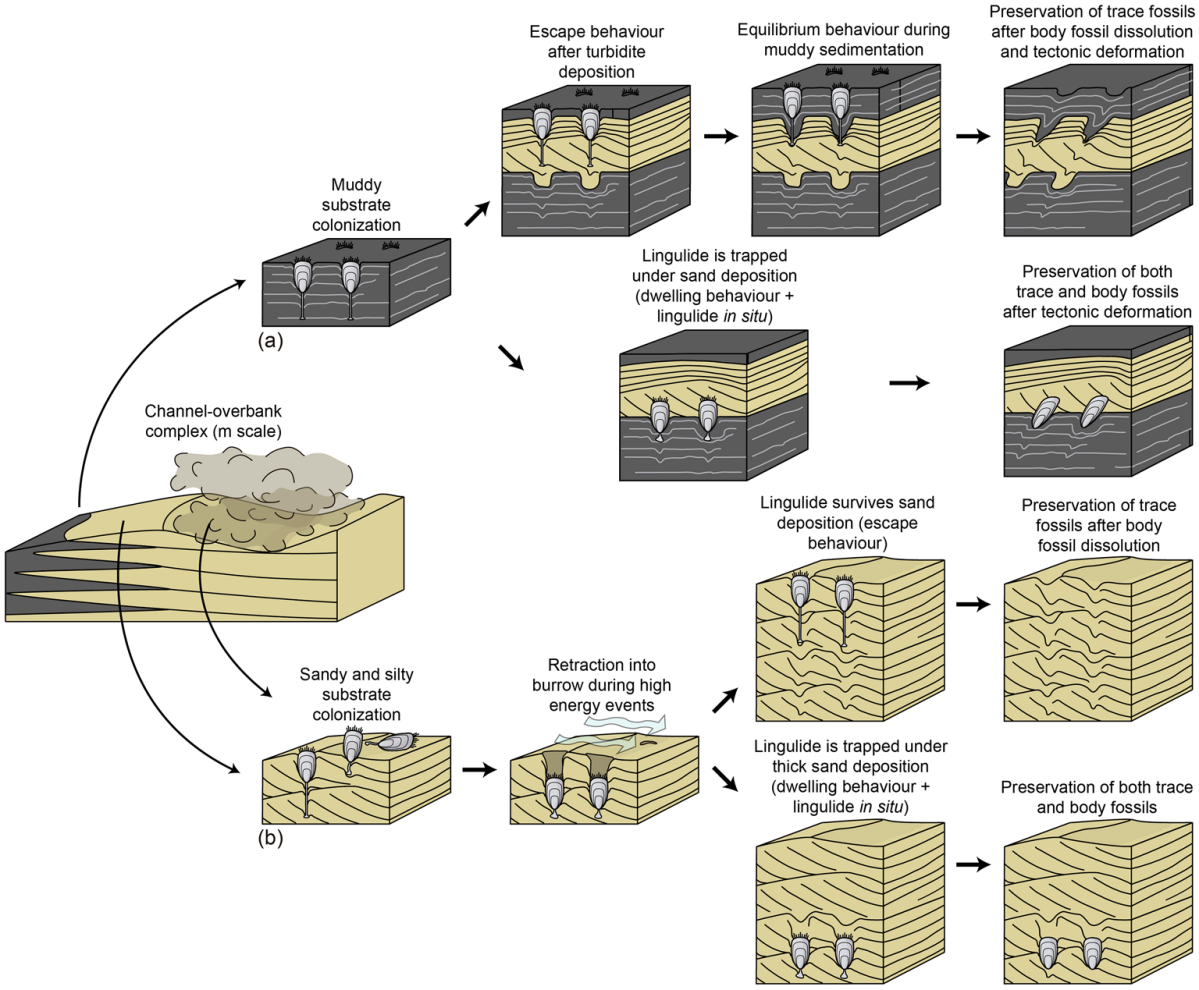
Taphonomic pathways and environmental distribution of *Lingulichnus verticalis* of the Agüeira Formation. (**a**) In heterolithic substrates of the overbank environment, escape behaviour took place during turbidite deposition, and equilibrium behaviour during hemipelagic, muddy sedimentation (more likely scenario). However, if the lingulide becomes trapped by the depositional event, dwelling structures and the lingulide shells may be preserved. Tectonic deformation generated burrow inclination in heterolithic deposits prone to shear. (**b**) In sandy and silty substrates of the overbank and channel environments, escape behaviour took place during turbidite deposition. However, thick sand deposition in such settings can trap organisms, generating preservation of dwelling structures and the lingulide shells. Created by M.P. in Adobe Illustrator CS6.

The studied specimens represent examples of three behaviours, namely escape, dwelling, and equilibrium (Fig. 6). The most common of these behaviours is escape, and it is inferred from the specimens in relatively thin-bedded siltstone. In these cases, the lingulides may have rapidly migrated upwards in response of turbiditic events to colonize the new sediment surface on top of the turbidites (Fig. 6a). Dwelling behaviour can be interpreted from the examples where the lingulides are preserved below the spreite (Figs. 5c,d, 6b). In such scenario, the organism must have re-colonized from the sediment surface above to its endobenthic position. At a later stage, the lingulide possibly migrated downwards to avoid being entrained and carried away by relatively high-energy currents, as modern lingulides can retract into their burrows if unfavorable environmental conditions occur^1,2^. The organism must have been unable to reposition its burrow close to the sediment–water interface and became buried, possibly due to high burial depths, which constitute an important parameter that determine organism survival rate^31^. Lingulides can escape burial of 10–30 cm of sediment in a few hours to a few days after deposition, probably using a combination of scissor, rotary sawing, sliding, and gaping motions with their valves^18,98,99^, yet survivorship might be reduced in some lingulides when buried deeper than 15 cm^100^. This agrees with the fact that this exceptional preservation was found in relatively thicker sandstone intervals. Finally, equilibrium behaviour can be interpreted from the existence of spreite in the mudstone intervals, during times of low sedimentation rate (Figs. 4f, 6a). Although fine-grained sediment usually shows insufficient shear strength that prevents pedicle attachment due to high water content^18^, the pedicle can extend far beneath the surface (up to 20 times the shell length^101^), where it may have been attached to buried sandstone or siltstone, or more compacted sediment below.

A typical lingulide burrow shows a spade shape due to the dwelling of the shell in the upper part, and the area where the pedicle is housed below. The pedicle anchors the brachiopod to the sediment, whereas the shell can move up and down within the upper burrow^2^. However, the pedicle burrow and spade morphology associated with *Lingulichnus* are only rarely preserved in the studied material (Fig. 3c). The rarity of pedicle burrow preservation is not clear, but it may be related to the rapid escape behaviour of the organism. Pedicle burrows of *Lingulichnus* seem to be preferentially preserved in parallel-laminated, heterolithic intervals associated with slow sedimentation (Zonneveld and Pemberton^30^, their Figure 5a,b). In fact, in *Lingulichnus* reported elsewhere, a few pedicle burrows show a laminated infill, indicating several instances of burrow filling during organism equilibrium (Zonneveld and Pemberton^30^, their Figure 5a,b). Hence, the pedicle burrow is preserved when only vertical movements of short extent were performed over long periods of time. In contrast, *Lingulichnus* associated with escape behaviour may not preserve the pedicle because of a rapid seafloor detachment (Zonneveld and Pemberton^30^, their Figure 5c).

The present specimens have similarities to *Scalichnus* Hanken et al*.*, 2001, and *Siphonichnus* Stanistreet et al*.*, 1980, two ichnogenera attributed to bivalves. In the first case, the existence of a spreite, muddy burrow boundary, and oval to circular burrow cross-sections may suggest similarities with *Scalichnus*. However, it is likely that the muddy burrow boundary represents part of the spreite, rather than a lining as in *Scalichnus.* This can be inferred by the typical occurrence of this burrow boundary in specimens towards the top of siltstone beds (Fig. 4e,f), indicating infill from the muddy intervals on top, and together with specimens with spreite in the same beds, strongly suggesting that this burrow boundary is best explained as a morphological variation of the spreite (Fig. 4g). Most important, the shape of *Scalichnus* significantly differs from the shape of the described trace fossils. *Scalichnus* is irregular in the lower part because of the bivalve burrowing technique (i.e. hydraulic burrowing by pumping a jet of water underneath the animal^102^), and laminated and irregular in the upper part due to sediment compression around the bivalve siphon^102^. This creates a typical sack- or bottle-like, irregular shape and a biodeformed mantle around the burrow, which contrasts with the tube- and spade-like morphology of the described trace fossil. In the case of *Siphonichnus*, the passively infilled core and a laminated outer mantle in the studied specimens are similar to features observed in this ichnogenus; however, the trace fossils from Asturias typically have an ovoid to elliptical burrow cross-section, whereas *Siphonichnus* has a circular cross-section^103,104^. As with *Scalichnus*, *Siphonichnus* has been attributed to bivalves^103,105^.

In the study area, U-shaped, vertical burrows attributable to *Arenicolites* have been described from the base of the formation, yet the morphology of this trace is different to the presently described trace fossil^83^. Outside of the Puerto de Vega location, in the Vega de Espinareda area (*ca*. 70 km south of the study area), inclined burrows attributable to *Skolithos* or *Cylindrichnus* have been described in a sand-rich succession, but the burrows have not been figured^84^. The possibility that these burrows are preservation variants of *Lingulichnus* cannot be discarded, but these specimens have not been found during our fieldwork and no further discussion is possible in the absence of illustrations. This possibility is in line with the suggestion of Zonneveld and Pemberton^30^ that many occurrences of *Lingulichnus* may have been misidentified as other vertical burrows.

## Discussion

The results of this analysis extend the bathymetric range of *Lingulichnus* to deep-marine environments and provide the oldest record of extensive colonization of proximal turbidite settings. *Lingulichnus* has only been described from shallow- and marginal-marine deposits in the fossil record. The deepest-water recording of *Lingulichnus* prior to this study corresponds to the Silurian Delorme Group of Canada, where this ichnotaxon occurs in carbonate shelf deposits (at the top of a shallowing-upward, slope to shelf sequence^106^).

Modern lingulides are generalists with respect to depth; hence, they are not only found in intertidal and shoreface settings, but also in deep-marine locations (thousands of meters deep^31,107^). Evaluation of the fossil record indicates that lingulides were established in all marine environments since the Cambrian, including abyssal depths^6^. In situ lingulides have been described from the Middle Ordovician deep-water carbonate slope deposits of the Table Head Group, Newfoundland, Canada^3^ and from basinal siliciclastic deposits of the Ludlow of the Welsh Borderland and Wales^108^. The reason for the disparity between the body and trace fossil records in Paleozoic deep marine environments is unknown. However, there is a lack of common terminology when referring to deep-marine abyssal environments between the paleontologic and sedimentologic communities. Lingulide shells are typical of black shales considered deep marine by paleontologists^109^. Yet, sedimentologists consider continental slope, turbidite fans, and abyssal plains located at a paleobathymetry in the order of thousands of meters as truly deep-marine environments. Hence, it may be that the known ancient lingulide distribution^6^ refers to shallower-water settings than the environments identified in the present contribution. Another possibility is that the Agüeira Formation represents shallow-water turbidites, yet none of the characteristics of the latter, such as presence of hummocky cross-stratification and combined-flow ripples reflecting oscillatory reworking of the event beds or direct association with nearshore deposits^110–113^, have been observed. In short, sedimentologic characteristics support the classic interpretation of the Agüeira Formation as deep marine^81–84^.

Spreite structures associated with *Lingulichnus* in the sandstone, siltstone, and mudstone indicate that the lingulides were adapted to deep-marine conditions and inhabited this location through long periods of time, instead of representing short-term colonization of an allochtonous community (Fig. 4f). Moreover, restriction of lingulide body fossils to the thicker sandstone intervals indicates that the organisms were usually buried during high sedimentation rate events, but they withstood the typical low-energy turbiditic events. Therefore, in the present case, lingulides may have preferred the overbank environment, where low-energy turbidity currents were conspicuous.

A likely hypothesis for the local abundance of lingulide trace fossils in deep-marine environments could be related to the existence of a continuous bottom current^106,114,115^. Current velocities above 25 cm/s promote suspension-feeding life strategies in the deep sea^114^, such as those observed in lingulides. Hence, deep-marine bottom currents providing abundant food particles in suspension could explain how lingulides became established in the studied turbidite system. Moreover, elevated levels of nutrients and organic matter in the currents may have excluded the benthic communities represented by the *Paleodictyon* subichnofacies of the *Nereites* Ichnofacies, which is typical of food-restricted deep-marine settings^49,68^. In the last decade, modern studies have recorded in detail the interaction of downslope turbidite systems with along-slope bottom currents, a mechanism that is common in most continental margins around the world^116^. Bottom currents rework previously deposited turbidites and deflect the diluted, low-density cloud associated with turbidite deposition in channels, generating preferential sedimentation towards the levee located downcurrent that produces channel-levee asymmetry^116,117^. Hence, overbank environments may have been affected by along-slope processes that could have provided suspended food particles from both turbidity clouds and adjacent areas to the lingulides.

The lingulides were probably transported to this deep-marine location by along-shore bottom currents or through turbiditic events associated with the channel. The fact that lingulides have planktotrophic larvae may have also facilitated dispersal into deep-marine settings^118,119^. Scarce body fossils and abundant trace fossils crosscutting the entire thickness of thin-bedded turbidites suggest a high success rate when escaping low-density turbidite events, indicating that sedimentation rate was not a strong stress factor for the organisms, at least in the overbank area. Substrate consistency does not seem to have affected the lingulide population, as equilibrium structures are also typical in the interbedded mudstone deposits. Data from modern environments is ambiguous with respect to substrate and the grain size in which lingulides prevail, with communities preferring both muddy and sandy substrates^1,2,21,120^. With respect to temperature, modern lingulides are restricted to equatorial and subtropical latitudes and low temperature is considered a stress factor^4^. However, the study area was in a high-latitude position^76^ and at a time of global cooling^121,122^, suggesting that lingulides were at once able to adapt to low water temperatures. Similarly, lingulide body and trace fossils were found in Upper Devonian lagoonal deposits^47^ and Late Carboniferous deltaic environments^41^ at high paleogeographic latitudes, supporting the idea of a greater ability to tolerate low temperatures and inhabit high latitudes earlier in their evolutionary history.

The finding of *Lingulichnus* in direct association with its producer has implications to unravel the early colonization of the deep sea. Whereas Cambrian deep-marine deposits are dominated by trace fossils reflecting the exploitation of microbial mats, Ordovician base of slope and basinal deposits show the appearance of ichnofaunas that are ethologically closer to those typical of the modern deep sea^58,60,123–127^. In this sense, a progressive increase in the proportion of farming and trapping structures with respect to those produced by deposit and detritus feeders is apparent through the Ordovician. These highly complex and elaborate structures, representative of K-selected population strategies, are dominant in the low-energy, distal areas of turbidite systems, particularly being preserved in frontal splay and levee deposits. However, colonization of high-energy, inner channelized segments of turbidite systems was relatively rare during the Ordovician^70,126,128^. Extensive colonization of these proximal zones took place during the Late Jurassic, when crustacean galleries assigned to *Ophiomorpha* became widespread and common in thick-bedded sandstone turbidites, recording the establishment of the *Ophiomorpha rudis* subichnofacies of the *Nereites* Ichnofacies^67,68^. Hence, the presence of high-density *Lingulichnus* in an Upper Ordovician turbidite channel-overbank complex represents the oldest evidence of widespread colonization in these high-energy proximal settings.

The opportunistic nature of lingulides makes them particularly suited for coping with the high energy and sedimentation rate of proximal turbidite systems. Lingulides are adapted to inhabit stressed environments such as intertidal settings and have been regarded as “disaster taxa” that can occupy vacant niches in the aftermath of extinction events^2,21–25^. The locally high densities and the monospecific nature of *Lingulichnus* suites in most of the studied deposits provide further evidence of r-selected populations, which sharply contrast with ichnofaunas of more distal turbidite settings that are illustrated by the archetypal elements of the *Nereites* Ichnofacies. A suspension-feeding strategy and the ability to burrow responding to erosion and rapid sedimentation events made lingulide populations able to thrive in these proximal settings, markedly differing from the trapping and farming strategies that are typical in more distal segments of the turbidite system.

## Materials and methods

The analysed interval corresponds to 35 m of the Agüeira Formation outcrops in the Frejulfe beach, located approximately 1 km west of Puerto de Vega, Asturias, northwest Spain (Fig. 1b). Standard facies analysis, based on the characterization of lithology, sedimentary structures, bed contacts, bed geometries and fossil content, provided the basis for paleoenvironmental interpretation. Field facies descriptions were refined through the study of thin sections under the petrographic microscope, allowing a precise lithologic characterization and detailed observation of trace fossil morphology and deformation features. Body fossil description and interpretation was based on outcrop observations. The ichnological analysis was mainly based on outcrop descriptions, whereas a few samples were taken and described with hand lens in the laboratory. Moreover, polished sections perpendicular and parallel to the trace fossils were made to best characterize their morphology. The description of trace fossils includes a detailed morphological assessment, measurements of burrow size and bioturbation index (BI), and interpretation of ethology.

### Supplementary Information

Supplementary Figure S1.

## Data Availability

The data that support the findings of this study are available from the corresponding author, M.P., upon reasonable request.
