# Supplementary material for: Deep-sea Ordovician lingulide brachiopods and their associated burrows suggest an early colonization of proximal turbidite systems
Source: Sci Rep. 2023 Dec 20;13:22753. doi: 10.1038/s41598-023-49875-8 (PMC10733332; doi:10.1038/s41598-023-49875-8)
Supplement: Supplementary file 1 — Supplementary Figure S1. [file 41598_2023_49875_MOESM1_ESM.docx]

Supplementary Figure S1


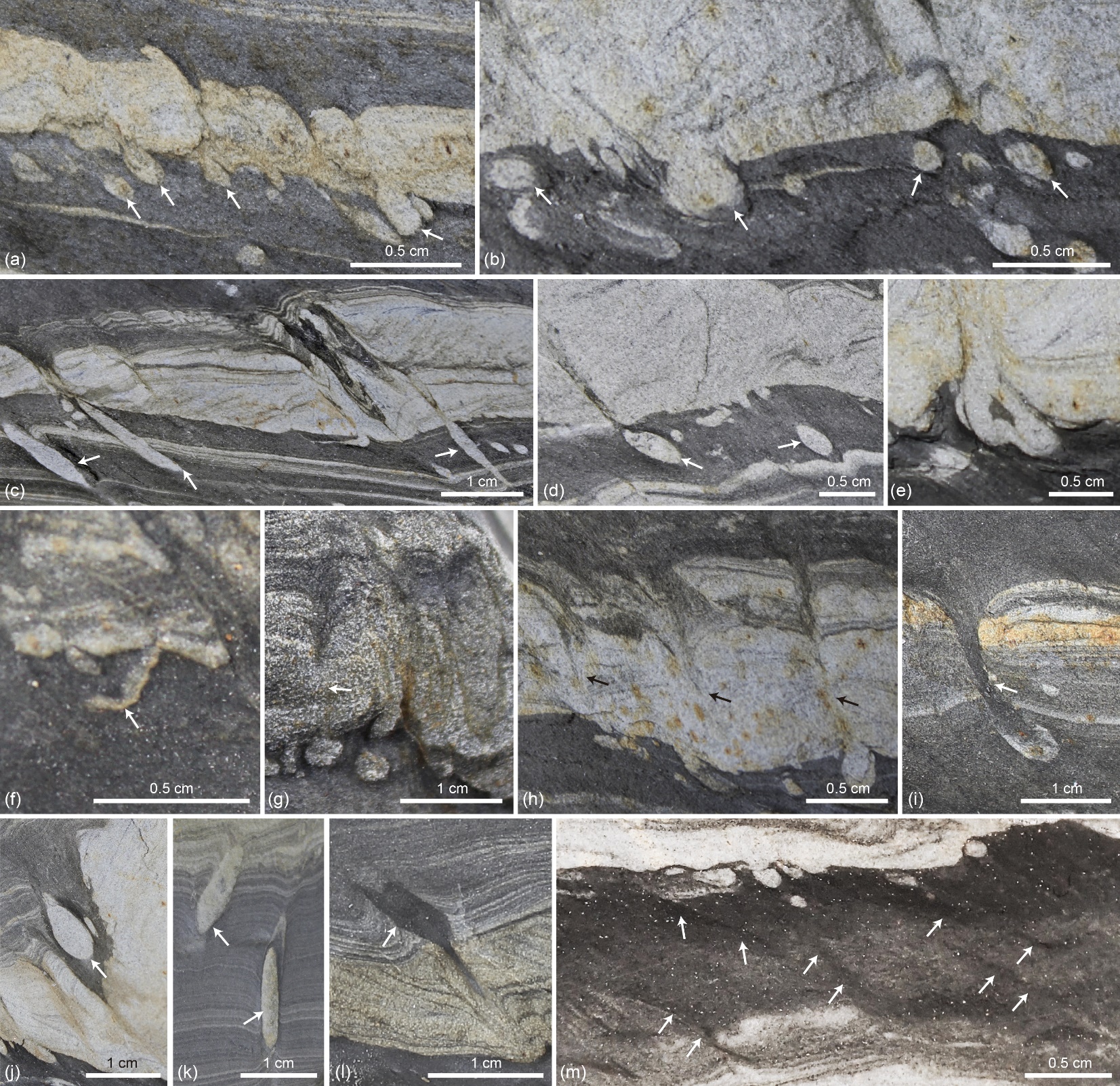


Detailed close-up photographs of *Lingulichnus verticalis* from the Upper Ordovician Agüeira Formation, Spain. (**a-f**) Lower structure located at the base of turbidite siltstone to sandstone (white arrows), showing rounded to ellipsoidal (a, b), tube (c), almond (d), upside-down heart (e), and “J” (f) morphologies. (**g-l**) Upper structure occurring within turbidite siltstone to sandstone (white and black arrows), displaying only the cone-in-cone spreite (g, h), or the spreite together with funnel (i), tube (j, k), and spade (l) burrows. (**m**) Equilibrium structures (white arrows) observed within mudstone deposits interbedded between turbidite siltstone to sandstone.
